# Oh baby! Motivation for healthy eating during parenthood transitions: a longitudinal examination with a theory of planned behavior perspective

**DOI:** 10.1186/1479-5868-10-88

**Published:** 2013-07-06

**Authors:** Rebecca L Bassett-Gunter, Ryna Levy-Milne, Patti Jean Naylor, Danielle Symons Downs, Cecilia Benoit, Darren E R Warburton, Chris M Blanchard, Ryan E Rhodes

**Affiliations:** 1 York University, Toronto, ON, Canada; 2 Behavioural Medicine Laboratory, Faculty of Education, University of Victoria, PO Box 3015 STN CSC, Victoria, BC V8W 3P1, Canada; 3 BC Cancer Agency, Vancouver, BC, Canada; 4 The Pennsylvania State University, State College, Pennsylvania, USA; 5 University of British Columbia, Vancouver, BC, Canada; 6 University, Halifax, Nova Scotia, Canada

**Keywords:** Theory of planned behaviour, Nutrition, Dietary behaviour, Parenthood

## Abstract

**Background:**

Transitioning to parenthood is a major life event that may impact parents’ personal lifestyles, yet there is an absence of theory-based research examining the impact of parenthood on motives for dietary behaviour. As a result, we are unaware of the social cognitive variables that predict eating behaviour among those transitioning to parenthood. The purpose of the study was to examine eating behaviour motives across 12 months within the framework of the theory of planned behavior (TPB) and compare these across groups of new parents, non-parents, and established parents.

**Methods:**

Non-parents (*n* = 92), new parents (*n* = 135), and established parents (*n* = 71) completed TPB questionnaires assessing attitudes, subjective norms, perceived behavioral control (PBC), and intentions and three day food records at baseline, and 6- and 12-months post-delivery (for parents) and 6- and 12-months post-baseline (for non-parents).

**Results:**

Repeated measures ANOVAs revealed that among men, new- and established-parents had greater intentions to eat healthy compared to non-parents, F(2) = 3.59, *p* = .03. Among women, established parents had greater intentions than new- and non-parents, F(2) = 5.33, *p* = .01. Among both men and women during the first 6-months post-delivery, new-parents experienced decreased PBC, whereas established parents experienced increased PBC. Overall, affective attitudes were the strongest predictor of intentions for men (β = 0.55, *p* < .001) and women (β = 0.38, *p* < .01). PBC predicted changes in fruit and vegetable consumption for men (β = 0.45, *p* = .02), and changes in fat consumption for men (β = −0.25, *p* = .03) and women (β = −.24, *p* < .05), regardless of parent status.

**Conclusion:**

The transition to parenthood for new and established parents may impact motivation for healthy eating, especially PBC within the framework of TPB. However, regardless of parental status, affective attitudes and PBC are critical antecedents of intentions and eating behaviour. Interventions should target affective attitudes and PBC to motivate healthy eating and may need to be intensified during parenthood.

## 

Consuming a diet high in fruits and vegetables and low in fat is important for maintaining a healthy body weight and preventing obesity-related diseases such as Type-2 diabetes, cancer, and cardiovascular disease [1]. Accordingly, Canada’s Food Guide [2] recommends that adults consume seven to 10 servings of fruits and vegetables each day to achieve overall health. Further, the guide recommends that no more than 35% of an adult’s daily caloric intake come from dietary fat consumption. Despite the well-established evidence that fruit and vegetable and fat consumption are important components of a healthy diet, approximately one half of Canadian adults fail to meet the respective guidelines [3]. There is a critical need to understand motivation for healthy eating in an effort to design and implement effective interventions [4]. Further, in order to be optimally effective, such interventions should be designed to target the most important determinants of healthy eating [4].

Various demographic groups may need targeting for nutritional intervention and new parents may be a critical group. Transitioning to parenthood is a major life event that may impact parents’ personal lifestyles, health behaviours and health-related attitudes [5-8]. Indeed, there is a growing body of evidence demonstrating an inverse relationship between parenthood and health behaviours such as physical activity for both mothers and fathers [9,10]. Parenthood presents increasing demands related to time, finances, fatigue, and childcare, which may interfere with motivation for various behaviours such as healthy eating [9,11]. For example, 98.6% of mothers reported that time commitments related to childcare were a barrier to physical activity [12]. Indeed, perceived barriers are a key predictor of intentions to exercise postpartum [7]. It is plausible that such increased demands could also reduce feelings of control over healthy eating. Although it has not been examined empirically, a lack of perceived control could be particularly evident among new parents who are faced with many unfamiliar demands. For some people however, the transition to parenthood may trigger a reappraisal of lifestyle and initiate psychological changes related to increased motivation for engaging in healthy behaviours [10]. For example, many parents have reported enhanced motivation to make positive lifestyle changes following parenthood transitions in order to create a healthy environment for their child(ren) [13].

Despite these possibilities, there is an absence of theory-based research examining the impact of parenthood on motives for eating behaviour, which has resulted in a general lack of awareness of how these variables may differ from that of the general population. The use of theoretical approaches to examining these predictors would be useful for identifying motives of new parents and whether they differ across time compared to non-parents and established parents (i.e., second-time parents). Gaining an understanding of theory-based predictors of dietary behaviours will facilitate the development of targeted interventions to improve, or prevent declines in, healthy eating among people transitioning to parenthood.

The theory of planned behaviour (TPB) [14] has been recommended as a valid framework for understanding dietary behaviours such as fruit and vegetable intake and fat consumption [15,16]. In fact, a recent systematic review suggests that the TPB is a preferred model for predicting fruit and vegetable consumption [4]. The TPB explains that behaviour is the result of one’s intentions (i.e., overall motivation) and perceptions of control over performance of the behaviour (i.e., perceived behavioural control; PBC). Intentions are determined by affective (i.e., emotion-based judgements about the behaviour) and instrumental attitudes (i.e., perceived benefits and costs of the behaviour), subjective norms (i.e., perceptions of significant others’ preferences about the behaviour), and PBC.

The TPB has been applied to the study of eating behaviours (e.g., fruit and vegetable and fat consumption) across various populations. A meta-analytic review found that, on average, the TPB explains 26.7% of the variance in dietary behaviour among the general population [16]. Within individual studies however, the amount of variance in eating behaviour explained by the TPB has ranged from 9% among adult patients at a health clinic [17] to 42% among obese and overweight individuals [18]. PBC has emerged as the strongest predictor of eating behaviours, while attitudes have demonstrated the strongest prediction of intentions [16].

Although TPB has not yet been applied to new parents and their eating behaviours, it is possible that the predictors may be unique [19]. For example, parenthood may bring about improvements in instrumental attitudes toward dietary behaviour as parents may reappraise the value or benefit of a healthy diet given the potential impact it may have on their children. For example, many parents believe it is wise to consume a healthy diet high in fruits and vegetables in order to act as role models for their children [13]. Accordingly, individuals transitioning to parenthood may experience an increase in intentions to engage in healthy eating. Alternatively, transitioning to parenthood may lead to deteriorations in PBC as parenthood may introduce or exacerbate barriers such as fatigue and a lack of time [13]. Among new mothers, self-efficacy and intentions predict postpartum exercise and food intake at one year postpartum [19]. Although this suggests that psychosocial variables such as PBC and intentions may be important predictors of healthy eating among new parents, at present, it is not known if transitioning to parenthood impacts TPB variables in relation to dietary behaviour or how the model predicts dietary behaviour among individuals experiencing parenthood transitions relative to the general population or non-parents. Furthermore, it is not known if the effects of parenthood transitions vary between new (i.e., first time parents) and established parents (i.e., second-time parents). There is some evidence to suggest that variables such as PBC may differ among first-time and established parents [8].

Thus, the purpose of this exploratory study was to examine changes in TPB components related to eating behaviour among first-time parents across their first year of parenthood and compare these predictors to established parents and non-parents. The following exploratory hypotheses were formed. It was hypothesized that compared to non-parents and established parents, new parents would have higher overall attitudes and intentions, and lower PBC toward healthy eating. Consistent with the TPB and previous research [17,20] it was hypothesized that attitudes and PBC would predict intentions to engage in healthy eating, with attitudes emerging as the strongest predictor. It was also hypothesized that intentions and PBC would predict changes in eating behaviour, with PBC being the strongest predictor [16]. Finally because of a lack of existing literature and evidence examinations of the moderating role of parent status were considered exploratory.

## Method

### Participants

**Figure 1:**
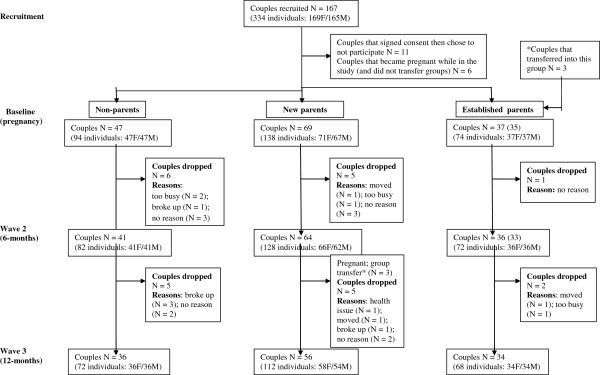
Participant recruitment and drop out.

Participants were men and women from the greater Victoria metropolitan area in British Columbia, Canada. Participants included non-parents (i.e., individuals with no children, not pregnant; *n* = 94), new parents (i.e., individuals expecting a first child at baseline; *n* = 138), and established parents (i.e., individuals expecting a second child at baseline; *n* = 74). These three demographic groups were chosen in order to evaluate potential unique group differences in motivation for healthy eating. All participants were part of a married or common-law couple (both partners of the relationship engaged as participants). Each couple was male–female with the exception of one female-female couple. Non-parents were significantly younger and had significantly lower household incomes than new parents and experienced parents. Recruitment of participants took place through in-person enlistment at baby fairs, word of mouth, and posters or pamphlets at parenting services venues (e.g., prenatal classes, baby stores, fitness centres, information centres, and clinics), recreation and community centres, libraries, healthcare centres and doctor and mid-wife offices, and coffee shops. Recruitment advertisements were also posted in local newspapers and online (e.g., Craigslist). Individuals became ineligible and were not included in the final sample if a) they (or their partners) experienced health complications due to pregnancy or birth (e.g., gestational diabetes, preeclampsia, bed-rest), b) they were non-parents who became pregnant, c) they were new parents who became pregnant a second time and chose to not continue as part of the established parents’ group. See Figure 1 for a flow diagram outlining participant recruitment and drop out. Table 1 displays the sample demographics.

**Table 1:** Demographic characteristics at baseline

**Characteristic**						
	**Non-parent (N = 94)**		**First-time parent (N = 139)**		**Established parent (N =74)**	
	**Women**	**Men**	**Women**	**Men**	**Women**	**Men**
Demographic profile						
Mean age	27	29.07	31.03	32.98	32.26	34.22
(SD)	(4.84)	(5.34)	(4.85)	(4.72)	(3.98)	(5.00)
% Visible minority	7.14	11.36	10.45	7.81	6.25	12.5
% Completed University	68.18	61.36	83.35	71.21	85.71	74.29
% > $75,000 Income	5.26	7.32	6.35	20.31	15.12	20.59
% Currently Employed	52.27	71.11	89.71	89.39	74.29	82.86

### Measures

#### Demographic variables

Participants indicated to which of the following categories they belonged at baseline: 1) non-parent, 2) new parent, or 3) established parent. Participants self-reported education by indicating the highest level of education they had completed: 1) ≤ 8th grade, 2) some high school, 3) high school diploma, 4) vocational school or some college, 5) college /university degree, 6) professional or graduate degree. Participant age and annual household income (dollars/year) were also self-reported.

#### Theory of planned behaviour variables

TPB variable items were in reference to “healthy eating each day” which was defined based on Canada’s Food Guide [2]. Participants were given examples of the Guide’s four food groups (i.e., grain products, milk products, fruits and vegetables, meat and alternatives), and the size and number of daily servings recommended. At baseline, expectant parents (i.e., new and established parents) were asked to answer each item in relation to “a typical week prior to pregnancy” whereas non-parents were asked to answer each item in relation to “a typical week over the next six months”. At six- and 12 month assessments all participants were asked to answer items within the context “during the next six months”. Each TPB item was anchored by a 5-point scale (1 *strongly disagree* to 5 *strongly agree*). These items were adapted from previous work examining the TPB in relation to eating behaviour [20,21] and were developed in accordance with the recommendations for measuring TPB variables [14].

#### Attitudes

Affective attitudes were measured with two items presented in the statement “It would be/would have been extremely 1) enjoyable and 2) fun for me to eat healthy each day”. Instrumental attitudes were measured with two items presented in the statement “It would be/would have been extremely 1) wise and 2) beneficial for me to eat healthy each day”. The items were averaged to calculate an overall affective attitudes score (Pearson’s r = .71; .65; .70 at baseline, wave two and wave three, respectively) and an overall instrumental attitudes score (Pearson’s r = .60; .76; .72 at baseline, wave two and wave three, respectively).

#### Subjective norms

Participants were instructed to answer questions considering what important people in their lives think they should do with regard to eating healthy each day. These items were presented in the statement “The most important people to me definitely 1) think/thought I should, 2) want/wanted me to, or 3) themselves eat healthy each day”. Participants then rated their agreement with the statement “The following people definitely would have thought/think I should eat healthy each day: 1) extended family, 2) friends, 3) health care workers 4) partner/spouse. The items were averaged to calculate an overall subjective norms score (Cronbach’s α = .76, .77, .76 at baseline, wave two and wave three, respectively).

#### Perceived behavioural control

Participants were instructed to answer questions regarding their confidence and/or control over eating healthy each day assuming they wanted to do so. First participants rated their agreement with the following three items: 1) I am/could have been completely confident, 2) I am/could have been in complete control, and 3) It will be/would have been extremely easy for me to…eat healthy each day. Next participants rated their agreement with five items presented in the statement “It would be/would have been extremely easy for me to eat healthy each day even if 1) I had limited time to prepare healthy food, 2) I didn’t have immediate access to healthy food, 3) I didn’t like the healthy food available, 4) I didn’t have enough money to buy healthy food, 5) I didn’t have a place to store or prepare healthy food”. The items were averaged to calculate an overall perceived behavioural control score (Cronbach’s α = .84, .80, .82 at baseline, wave two and wave three, respectively).

#### Intentions to engage in healthy eating

Participants rated their agreement with two items regarding their intentions to eat healthy each day for the next six months. The statements were presented as “I am 1) definitely motivated and 2) extremely determined to eat healthy each day”. The items were averaged to calculate an overall intentions score (Pearson’s *r* = .72, .72, .80 at baseline, wave two and wave three, respectively).

#### Eating behaviour

Participants completed a three day food record indicating all foods consumed over the course of three consecutive days (i.e., two weekdays and one weekend day). Participants were instructed to record everything they consumed and to maintain normal eating and drinking habits over the course of the recall days. The questionnaire included detailed instructions, examples, and measurement guides. The three day food record has been found to be a valid and reliable measure of diet quality among Canadian adults [22]. Average daily servings for each of the four food groups (i.e., grain products, milk products, fruits and vegetables, meat and alternatives) were calculated based on food frequency records and serving sizes outlined by Canada’s food guide [2]. Participants were coded as *Meeting* the guidelines if the number of daily servings for each food group were within the recommended ranges (e.g., 2–4 servings of milk products). Participants were coded as *Not Meeting* the guidelines if the number of daily servings for any food group was above or below the recommended range.

### Procedure

A longitudinal study design was used and included three data collection waves: 1) Baseline; following recruitment and during pregnancy for new and established parents, 2) Wave Two; 6 months following baseline for non-parents; 6 months post-delivery for new and established parents, and 3) Wave Three; 12 months following baseline for non-parents; 12 months post-delivery for new and established parents. Demographic variables were assessed at baseline. TPB and dietary behaviour were assessed at baseline, wave two, and wave three. Participants were mailed a package at each of the three data collection points. The package included the appropriate questionnaires and a postage-paid return envelope. Each participant received a t-shirt after completing the baseline questionnaire, as well as a $25 honorarium, which increased by $5 for each subsequent wave of data collection returned. Data collection was part of a larger study. The study protocol was approved by the institution’s Human Research and Ethics Review Board, and all participants provided informed consent.

### Data analyses

#### Potential covariates

Data for men and women were analyzed separately due to the dyadic nature of the data. Demographic variables such as age, income, and education may be associated with dietary behaviours [18]. The following analyses were conducted to examine these potential covariates of intentions and dietary behaviour; 1) Pearson’s correlations were calculated for age and income, 2) a Spearman’s rank correlation was calculated for education, and 3) an ANOVA was calculated for primary method of feeding. All covariates identified were controlled for in the appropriate analyses.

#### Changes in TPB variables during parenthood transitions

Repeated-measures ANOVAs were calculated to examine changes in attitudes, subjective norms, PBC, and intentions by parent status across the three data collection waves (i.e., baseline, 6-months, 12-months).

#### TPB variables predicting intentions to eat healthy; parent status as a possible moderator

Hierarchical linear regression models were calculated with intentions as the dependent variable. The intentions data were skewed and submitted to a square root transformation to normalize the distribution. Attitudes, subjective norms, and PBC variables were zero-centred [23] and entered on the first step of each regression in addition to parent status. Interaction terms (i.e., product of parent status multiplied by each centred TPB variable) were entered on the second step of each regression using a step-wise approach. In the presence of a significant interaction (*p* < .05), post hoc analyses were conducted to determine the form of the interaction. Separate regression equations were calculated for each parent status group, in which intentions were regressed onto the TPB variable from the significant interaction term. Predicted levels of the TPB variable were then calculated and plotted using the mean intention score, and scores one standard deviation above and below the mean [24].

#### TPB variables predicting changes in eating behaviour; parent status as a possible moderator

The original data analysis plan involved the calculation of logistic regression models examining TPB variables as predictors of *Meeting* versus *Not Meeting* Canada’s Food Guide [2] recommendations. However, there was limited variability when treating eating behaviour as a binary variable. For example, less than five percent of the sample were *meeting* the recommended guidelines across all four food groups at wave 2. Accordingly, two subsequent indicators of eating behaviour were calculated as continuous variables.

#### Average daily fruit and vegetable servings

Average daily servings of fruits and vegetables were calculated based on food frequency records and serving sizes outlined by Canada’s food guide [2]. Fruit and vegetable consumption is an important component of healthy dietary behaviour outlined in Canada’s food guide recommendations and is a common indicator of “healthy eating” within TPB research e.g., [17,18,20,25,26].

#### Average daily fat consumption

Average daily grams of fat consumed were calculated based on food frequency records. Fat consumption is an important component of healthy eating behaviour and has been considered an indicator of “healthy eating” within previous TPB research [17]. Hierarchical linear regression models were calculated with a) fruit and vegetable consumption and b) fat consumption as the dependent variables. These data were skewed and submitted to square root transformations in order to normalize the distribution. In order to examine changes in eating behaviour, baseline (or 6-month) scores of the dependent variable were entered on step one of each regression. Any significant covariates were also entered on step one. Parent status, PBC, and intentions were entered on step two. Affective and instrumental attitudes, and subjective norms were entered on step three. Interaction terms (the product of parent status multiplied by each centred TPB variable) were entered on step four using a step-wise approach (exclusive to step four). Given the large number of potential interaction effects and the subsequent likelihood of multicollinearity, the step-wise approach was deemed most appropriate for testing interaction terms in this exploratory research question. This approach has been used in previous research e.g., [27,28]. In the presence of a significant interaction (*p* < .05), post hoc analyses were conducted to determine the form of the interaction; separate regression equations were calculated for each parent status group, in which the dietary behaviour variable was regressed onto the TPB variable from the significant interaction term. Predicted levels of the TPB variable were then calculated and plotted using the mean dietary behaviour score, and scores one standard deviation above and below the mean.

## Results

### Potential covariates

Age (Men *M* age = 32.1 years; women *M* age = 30.1 years) and household income were not significant covariates of any dependent variable. For men, education was significantly related to baseline intentions (Spearman’s r = 0.18, *p* = 0.03) and 12-month fat consumption (Spearman’s r = 0.20, *p* = 0.04). Analyses involving these dependent variables were adjusted for participants’ education.

### Group differences and changes in TPB variables during parenthood transitions

Additional file 1: Table S1 displays the results of the repeated measures ANOVAs examining group differences and changes in TPB variables. Significant main effects for condition indicated that among men, new parents had greater intentions than non-parents and established parents, F(2) = 3.59, *p* = .03. Among women, new parents had stronger instrumental attitudes, F(2) = 3.03, *p* = .05, and intentions, F(2) = 5.33, *p* = .01, compared to established parents. Non-parents also had stronger instrumental attitudes and intentions compared to established parents. Significant time by condition interaction effects were found for PBC among men, F(4,202) = 3.79, *p* = .01, and women, F(4,210) = 9.64, *p* < .001. Post hoc analyses indicated that there was a significant decrease in PBC from baseline to six-months among new parents, and a significant increase in PBC from baseline to six-months among established parents.

### TPB variables predicting intentions to eat healthy; parent status as a possible moderator

Table 2 displays results of the final model^a^ of hierarchical regression analyses examining predictors of intentions. Affective attitudes (AA) and PBC were significant predictors of intentions among both men, (AA: β = 0.55, *p* < .001; PBC: β = 0.15, *p* = .02) and women (AA: β = 0.38, *p* < .01; PBC: β = 0.27, *p* = .02). Subjective norms predicted intentions among men (β = 0.16, *p* = .01), whereas a significant subjective norms by parent status interaction was observed for women (β = 0.32, *p* = .01). Post-hoc analyses indicated that subjective norms were a significant predictor of intentions among new (β = 0.44, *p* < .001) and established mothers (β = 0.55, *p* < .001), but not among non-parent women.

**Table 2:** Hierarchical regression of TPB variables predicting intentions to eat healthy

	**R**^**2**^	**R**^**2**^**Δ**	***p***	**β**
Women (N = 144)				
Final model	0.41	0.03	0.11	
Parent status				−0.22^**^
Affective attitudes				0.38^**^
Instrumental Attitudes				0.22^†^
Subjective norms				−0.12
PBC				0.27^*^
Affective attitudes x parent status				<0.01
Instrumental attitudes x parent status				−0.17
Subjective norms x parent status				0.32^*^
PBC x parent status				−0.14
Men (N = 145)				
Final model	0.54	0.53	< 0.01	
Parent status				−0.05
Affective attitudes				0.55^**^
Instrumental attitudes				0.09
Subjective norms				0.16^*^
PBC				0.15^*^

Note. Model for male participants was adjusted for education. **p < .01, *p < .05, †p < .10.

### TPB variables predicting changes in eating behaviour; parent status as a possible moderator

#### Changes in fruit and vegetable consumption

Table 3 (women) and Table 4 (men) display results of the final models^a^ of hierarchical regression analyses examining predictors of changes in fruit and vegetable consumption. PBC was the only significant predictor of changes in fruit and vegetable consumption from baseline to six-months (β = 0.45, *p* = .02) among men. Instrumental attitudes (β = 0.25, *p* = .02) and parent status (β = −0.26, *p* < .01) predicted changes in fruit and vegetable consumption from 6 to 12-months among men. Among women, a significant main effect of affective attitudes (β = 0.35, p = .01) was superseded by an affective attitude by parent status interaction (β = −.40, *p* = .03). Post hoc analyses indicated that affective attitudes were a significant predictor of changes in fruit in vegetable consumption from baseline to six-months among non-parent women only (β = 0.58, *p* < .001). There were no significant predictors of changes in fruit and vegetable consumption from 6 to 12-months among women.

**Table 3:** **Hierarchical regression of TPB variables predicting fruit** &**vegetable consumption for women**

**Six-month (N = 116)**	**R**^**2**^	**R**^**2**^**Δ**	***P***	**β**	**12-month (N = 109)**	**R**^**2**^	**R**^**2**^**Δ**	***p***	**β**
Final model	0.21	0.06	<.001		Final model	0.17	0.01	0.68	
Baseline fruit & vegetable				0.42^**^	6-month fruit & vegetable				0.39^**^
Parent status				−0.05	Parent status				−0.02
PBC				0.07	PBC				0.15
Intentions to eat healthy				0.03	Intentions to eat healthy				0.02
Affective attitudes				0.35^*^	Affective attitudes				0.01
Instrumental attitudes				0.14	Instrumental attitudes				0.02
Subjective norms				−0.08	Subjective norms				−0.12
Affective attitudes x parent status				−0.40^*^					

Note. **p < .01, *p < .05.

**Table 4:** **Hierarchical regression of TPB variables predicting fruit** &**vegetable consumption for men**

**Six-month (N = 119)**	**R**^**2**^	**R**^**2**^**Δ**	***p***	**β**	**12-month (N = 101)**	**R**^**2**^	**R**^**2**^**Δ**	***p***	**β**
Final model	0.09	0.19	0.06		Final model	0.19	0.08	0.05	
Baseline fruit & vegetable				0.20^*^	6 − month fruit & vegetable				0.38^*^
Parent status				0.05	Parent status				−0.26^*^
PBC				0.45^*^	PBC				−0.02
Intentions to eat healthy				0.15	Intentions to eat healthy				0.09
Affective attitudes				−0.01	Affective attitudes				−0.21^†^
Instrumental attitudes				0.25	Instrumental attitudes				0.25^*^
Subjective norms				−0.19	Subjective norms				−0.05
PBC x parent status				−0.27					
Intentions x parent status				0.05					
Affective Attitudes x parent status				0.07					
Instrumental attitudes x parent status				0.06					
Subjective norms x parent status				0.20					

Note. *p < .05, †p < .10.

#### Changes in fat consumption

Table 5 (women) and Table 6 (men) display results of the final models^a^ of hierarchical regression analyses examining predictors of changes in fat consumption. Among women, a significant PBC by parent status interaction was detected (β = −.28, *p* = .05). Post hoc analyses indicated that PBC was a significant (positive) predictor of changes in fat consumption from baseline to six-months among non-parent women only (β = 0.34, *p* = .04) with higher PBC predicting higher fat consumption. PBC was the only significant predictor of changes in fat consumption from 6 to 12-months among women (β = −.24, *p* < .05) with higher PBC predicting lower fat consumption. Among men, PBC was a significant positive predictor of changes in fat consumption from baseline to six-months (β = 0.27, *p* = .008) and a significant negative predictor of changes in fat consumption from 6 to 12-months (β = −0.25, *p* = .03).

**Table 5:** Hierarchical regression of TPB variables predicting fat consumption for women

**Six-month (N = 121)**	**R**^**2**^	**R**^**2**^**Δ**	***p***	**β**	**12-month (N = 108)**	**R**^**2**^	**R**^**2**^**Δ**	***p***	**β**
Final model	0.07	0.03	0.05		Final model	0.14	0.02	0.44	
Baseline fat				0.19*	6-month fat				0.38^**^
Parent status				0.12	Parent status				0.11
PBC				0.22	PBC				−0.24^*^
Intentions to eat healthy				−0.11	Intentions to Eat healthy				0.17
Affective attitudes				0.07	Affective attitudes				−0.13
Instrumental attitudes				0.02	Instrumental attitudes				0.11
Subjective norms				0.11	Subjective norms				0.07
PBC x parent status				−0.28^*^					

Note. **p < .01, *p < .05.

**Table 6:** Hierarchical regression of TPB variables predicting fat consumption for men

**Six-month (N = 119)**	**R**^**2**^	**R**^**2**^**Δ**	***p***	**β**	**12-month (N = 101)**	**R**^**2**^	**R**^**2**^**Δ**	***p***	***β***
Final model	0.06	0.01	0.74		Final model	0.16	0.01	0.66	
Baseline fat				0.24^**^	6-month fat				0.38^**^
Parent status				0.04	Parent status				0.15
PBC				0.27^**^	PBC				−0.25^*^
Intentions to eat healthy				0.05	Intentions to eat healthy				0.12
Affective attitudes				−0.13	Affective attitudes				−0.08
Instrumental attitudes				0.02	Instrumental attitudes				0.07
Subjective norms				−0.05	Subjective norms				0.09

Note. 12-Month model adjusted for participants’ education. **p < .01, *p < .05.

## Discussion

The purpose of the current study was to examine eating behaviour across time within the framework of the TPB and compare social cognitive motives within a group of new parents, non-parents and established parents. To our knowledge this is one of the first studies to longitudinally examine eating behaviour motivation within a theoretical framework among men and women experiencing parenthood transitions.

### Group differences and changes in TPB variables during parenthood transitions

It was hypothesized that compared to non-parents and established parents, new parents would have greater attitudes and intentions, and lower PBC toward healthy eating. In partial support of our hypothesis, intentions to eat healthy varied by parent status for both men and women. For men, new and established parents had greater intentions to eat healthy compared to non-parents suggesting that parenthood may have triggered an increased motivation for healthy eating. Consistent with previous research, parenthood may facilitate a reappraisal of lifestyle and initiate psychological changes related to increased motivation for engaging in behaviours such as healthy eating [10]. Some fathers may realize that they are role models for their children’s dietary behaviour [29] and thus experience increased motivation to eat healthy with the intention of positively impacting their children’s behaviour.

For women, established parents had lower intentions to eat healthy compared to new parents and non-parents suggesting a negative impact of multiple children on women’s motivation. Many women may be responsible for eating behaviour-related chores within a household (e.g., grocery shopping, meal planning and preparation) [30]. Some mothers may find that focusing time and energy on preparing healthy meals for multiple children leads to subsequent decreases in motivation for their own dietary behaviours. Perhaps for many mothers having more than one child may result in increased demands related to time, finances, fatigue, and social support, which may interfere with motivation for healthy eating [31,32]. Further research should examine the effects of multiple children on women’s experiences related to parenting.

For both men and women, changes in PBC varied by parent status. Consistent with our hypothesis, new parents experienced a decrease in PBC during the six-month post-delivery period. As has been found in the physical activity domain, this decrease in feelings of confidence and control regarding healthy dietary behaviour may not be surprising as new mothers and fathers face the demands of parenthood that may impact health behaviour motivation (e.g., fatigue, limited time) [9]. New parents may benefit from targeted interventions that aim to enhance PBC during the first six months. Alternatively, established parents experienced increased PBC during the six months following the birth of their second child. Based on their experience with the birth of their first child, many established parents may have lacked confidence and feelings of control as they anticipated the demands of a new baby. However, after the arrival of a second child, these parents may have drawn on previous experience to better cope. PBC as a predictor of exercise behaviour has also been found to vary among first time and established parents [8].

### TPB variables predicting intentions to eat healthy; parent status as a possible moderator

It was hypothesized that attitudes and PBC would predict intentions to engage in healthy eating, with attitudes emerging as the strongest predictor. In support of our hypothesis, affective attitudes, instrumental attitudes, and PBC were significant predictors of intentions to eat healthy among men and women. Consistent with previous research [16], affective attitudes were the strongest predictor of intentions for men (large effect size) and women (medium effect size), regardless of parent status. Regardless of parenthood status, it would seem that affective attitudes should remain the focus of interventions targeting motivation for healthy eating for all adults [31]. For example, health messages that emphasis the enjoyment one may experience from healthy eating may be useful to enhance dietary behaviour motivation.

Interestingly, subjective norms were a predictor of intentions to eat healthy among women who were new parents (medium-large effect size) and established parents (large effect size) but not fathers. Previous research has demonstrated that subjective norms do not generally predict intentions to eat healthy among adults [17,20]. However, women who are parents may rely on the perceived normative beliefs of important others (e.g., health care worker, spouse) when forming their intentions to eat healthy. There is evidence that subjective norms are important in determining other behaviours such as breastfeeding among mothers [33,34]. This tendency to rely on the perceived beliefs of important others may represent a unique characteristic that warrants targeted intervention to enhance subjective norms (and thus maximize dietary behaviour motivation) among new and established mothers.

### TPB variables predicting changes in eating behaviour; parent status as a possible moderator

Contrary to hypothesis, intentions to eat healthy did not predict changes in dietary behaviour for men or women, regardless of parent status. This was unexpected given that intentions are a key predictor of behaviour within the TPB and have been found to predict dietary behaviour in previous research [17,20,26]. However, this finding highlights to the intention-behaviour gap [35] often observed when considering health behaviours within the framework of the TPB and speaks to the relatively weak evidence for intentions as an actual antecedent of behaviour [35,36]. Future research should continue to examine motivation for dietary behaviour while also investigating interventions to reduce the intention-behaviour gap. Further, future research should examine other theoretical constructs such as planning, self-regulation, habit, and goal-conflict that may predict health behaviours [37-41].

In support of our hypothesis, however, PBC was a significant predictor of changes in eating behaviour under certain circumstances. For men, PBC was a positive predictor of changes in fruit and vegetable consumption at six months suggesting that men who had greater PBC increased their fruit and vegetable consumption (large effect size). For both men and women, PBC was a negative predictor of changes in fat consumption at 12 months suggesting that those who had greater PBC decreased their fat consumption (small-medium effect size). The relationship between PBC and changes in eating behaviour did not vary by parent status suggesting that PBC is an important predictor for non-parents, new parents, and established parents. Indeed, evidence from a meta-analysis suggests that PBC is the most important predictor of adult eating behaviour [16]. PBC should be a main focus of TPB-based dietary interventions for all adults. Despite having positive attitudes toward healthy eating and intentions to eat healthy, the results of the current study suggest that PBC is critical for implementing dietary behaviour change. Although parent status did not generally play a significant role in predicting changes in dietary behaviour, the observed decrease in PBC among new parents during the first six months post-partum suggests that new parents particularly may benefit from interventions that enhance PBC for healthy eating.

### Limitations and future directions

Despite the numerous strengths of this work including the theoretical framework and longitudinal design, there are limitations which warrant mention. As a result of the low variability when eating behaviour was classified as a binary variable, we used alternative sub-classifications, which may have resulted in reduced correspondence framing between the TPB and the dependent variable. In order to assess variables pre-pregnancy, parents recalled a typical week prior to pregnancy. Alternatively, variables were assessed in the traditional manner for non-parents (i.e., non-parents predicted a typical week in the next six-months). This methodological difference may have introduced a bias in reference to time and could be a potential limitation. Further, there could be additional biases between the samples (i.e., beyond parent status) that may account for some of the group-differences presented in Additional file 1: Table S1. Finally, the length of the study design permitted measurement of eating behaviour motivation for only 12-months beyond initial parenthood. Accordingly, changes in behaviour motivation beyond 12-months post-partum were not assessed. Future longitudinal research is necessary to further understand the long-term impact of parenthood on eating behaviour motivation.

## Conclusion

In summary, this is one of the first known studies to examine eating behaviour across time within the framework of the TPB and comparing motives within a group of new parents, non-parents, and established parents. New and established parents may experience changes in motivation for healthy eating, especially PBC. However, the same critical antecedents of affective attitude and PBC predict eating behaviour regardless of parent status. This suggests that TPB interventions are appropriate but may need to be intensified during parenthood transitions.

## Endnote

^a^See supplementary material (Additional file 2) for all models of the hierarchical regression analyses.

## Competing interests

The authors declare that they have no competing interests.

## Authors’ contributions

RBG carried out data analyses and was the primary author responsible for manuscript preparation. RLM, PJN, DSD, DW, CB and RR conceived and developed the study and design, as well as edited the manuscript. RR also consulted on the data analyses and directed data collection. All authors read and approved the final manuscript.

## Supplementary Material

Additional file 1 Table S1 Repeated Measures ANOVA of TPB Variables by Parent Status. Click here for file

Additional file 2 Table S2 Hierarchical Regression of TPB Variables Predicting Intentions to Eat Healthy. **Table S3.** Hierarchical Regression of TPB Variables Predicting Fruit & Vegetable Consumption for Women. **Table S4.** Hierarchical Regression of TPB Variables Predicting Fruit & Vegetable Consumption for Men. **Table S5.** Hierarchical Regression of TPB Variables Predicting Fat Consumption for Women. **Table S6.** Hierarchical Regression of TPB Variables Predicting Fat Consumption for Men. Click here for file
