# Supplementary material for: Oh baby! Motivation for healthy eating during parenthood transitions: a longitudinal examination with a theory of planned behavior perspective
Source: Int J Behav Nutr Phys Act. 2013 Jul 6;10:88. doi: 10.1186/1479-5868-10-88 (PMC3706269; doi:10.1186/1479-5868-10-88)
Supplement: Additional file 1 Table S1 — Repeated Measures ANOVA of TPB Variables by Parent Status. [file 1479-5868-10-88-S1.doc]

Table S1. Repeated Measures ANOVA of TPB Variables by Parent Status

|  | Baseline | Six-Month | 12-Month |  | | |
| --- | --- | --- | --- | --- | --- | --- |
|  | M (SD) | M(SD) | M (SD) | Time | Condition | Time x Condition |
|  |  |  |  | F (partial η^2^) | F (partial η^2^) | F (partial η^2^) |
| **Affective Attitudes** |  |  |  |  |  |  |
| Women |  |  |  | 1.34(.01) | 2.51(.05)^†^ | 1.18(.02) |
| Non-parents (n=31) | 3.91(.76) | 4.00(.72) | 3.92(.76) |  |  |  |
| New parents (n=47) | 4.04(.71) | 4.01(.79) | 3.99(.82) |  |  |  |
| Established parents (n=31)^b^ | 3.52(.63) | 3.81(.72) | 3.74(.89) |  |  |  |
| Men |  |  |  | 6.43(.06)** | 1.43(.03) | 0.49(.01) |
| Non-parents (n=30) | 3.50(.86) | 3.68(.89) | 3.68(.75) |  |  |  |
| New parents (n=44) | 3.74(.69) | 3.90(.65) | 3.91(.75) |  |  |  |
| Established parents (n30) | 3.43(.82) | 3.82(.77) | 3.68(.79) |  |  |  |
| **Instrumental Attitudes** |  |  |  |  |  |  |
| Women |  |  |  | 0.06(.001) | 3.03(.05)* | 0.46(.01) |
| Non-parents (n=31) | 4.87(.39) | 4.87(.32) | 4.82(.35) |  |  |  |
| New parents (n=49) | 4.84(.33) | 4.86(.34) | 4.90(.34) |  |  |  |
| Established parents (n=31)^a,c^ | 4.74(.41) | 4.73(.44) | 4.69(.46) |  |  |  |
| Men |  |  |  | 0.22(.002) | 1.46(.03) | 0.34(.01) |
| Non-parents (n=32) | 4.59(.50) | 4.61(.62) | 4.59(.47) |  |  |  |
| New parents (n=46) | 4.74(.42) | 4.71(.45) | 4.73(.48) |  |  |  |
| Established parents (n=30) | 4.52(.59) | 4.60(.62) | 4.63(.51) |  |  |  |
| **Subjective Norms** |  |  |  |  |  |  |
| Women |  |  |  | 1.02 (.01) | 1.62(.03) | 0.62(.01) |
| Non-parents (n=30) | 3.93(.47) | 3.94(.63) | 4.01(.53) |  |  |  |
| New parents (n=47) | 3.98(.48) | 4.02(.40) | 4.09(.43) |  |  |  |
| Established parents (n=31) | 3.87(.48) | 3.87(.50) | 3.84(.47) |  |  |  |
| Men |  |  |  | 4.90 (.09)** | 0.20(<.01) | 0.60(.01) |
| Non-parents (n=30) | 3.73(.38) | 3.92(.48) | 3.90(.53) |  |  |  |
| New parents (n=47) | 3.87(.53) | 3.91(.43) | 3.92(.42) |  |  |  |
| Established parents (n=31) | 3.74(.49) | 3.92(.52) | 3.92(.49) |  |  |  |
| **Perceived Behavioural Control** |  |  |  |  |  |  |
| Women |  |  |  | 7.00(.06)** | 2.32(.04)^†^ | 9.64(.16)** |
| Non-parents (n=31) | 3.34(.80) | 3.53(.67) | 3.56(.75) |  |  |  |
| New parents (n=47)^a,c^ | 3.34(.69) | 3.06(.72) | 3.29(.79) |  |  |  |
| Established parents (n=30) | 3.15(.76) | 3.74(.55) | 3.63(.66) |  |  |  |
| Men |  |  |  | 5.56(.10)** | 2.32(.04)^†^ | 3.79(.07)** |
| Non-parents (n=31) | 3.30(.66) | 3.45(.71) | 3.34(.71) |  |  |  |
| New parents (n=47)^c^ | 3.25(.70) | 3.20(.64) | 3.28(.70) |  |  |  |
| Established parents (n=30) | 3.12(.68) | 3.70(.60) | 3.67(.54) |  |  |  |
| **Intentions** |  |  |  |  |  |  |
| Women |  |  |  | 6.11(.05)** | 5.33(.09)** | 0.66(.01) |
| Non-parents (n=31) | 4.08(.73) | 4.19(.80) | 4.32(.77) |  |  |  |
| New parents (n=47) | 3.94(.82) | 4.14(.64) | 4.06(.77) |  |  |  |
| Established parents (n=31)^a,b^ | 3.52(.60) | 3.84(.60) | 3.76(.85) |  |  |  |
| Men |  |  |  | 0.33(.01) | 3.59(.06)* | 0.63(.01) |
| Non-parents (n=32) | 3.70(.76) | 3.72(.78) | 3.75(.72) |  |  |  |
| New parents (n=47)^a,c^ | 3.96(.64) | 3.85(.71) | 4.01(.74) |  |  |  |
| Established parents (n=30) | 3.55(.65) | 3.68(.52) | 3.62(.63) |  |  |  |
| Note. ^a^ significantly different from non-parents. ^b^ significantly different from new parents. ^c^ significantly different from established parents. **p<.01, *p<.05, †p<.10 | | | | | |  |
