# Supplementary material for: Oh baby! Motivation for healthy eating during parenthood transitions: a longitudinal examination with a theory of planned behavior perspective
Source: Int J Behav Nutr Phys Act. 2013 Jul 6;10:88. doi: 10.1186/1479-5868-10-88 (PMC3706269; doi:10.1186/1479-5868-10-88)
Supplement: Additional file 2 Table S2 — Hierarchical Regression of TPB Variables Predicting Intentions to Eat Healthy. Table S3. Hierarchical Regression of TPB Variables Predicting Fruit & Vegetable Consumption for Women. Table S4. Hierarchical Regression of TPB Variables Predicting Fruit & Vegetable Consumption for Men. Table S5. Hierarchical Regression of TPB Variables Predicting Fat Consumption for Women. Table S6. Hierarchical Regression of TPB Variables Predicting Fat Consumption for Men. [file 1479-5868-10-88-S2.doc]

Table S2. Hierarchical Regression of TPB Variables Predicting Intentions to Eat Healthy

|  | R^2^ | R^2^Δ | *p* | β |
| --- | --- | --- | --- | --- |
| Women (N = 144) |  |  |  |  |
| Model 1 | 0.39 | 0.42 | <0.01 |  |
| Parent Status |  |  |  | -0.22^**^ |
| Affective Attitudes |  |  |  | 0.38^**^ |
| Instrumental Attitudes |  |  |  | 0.10 |
| Subjective Norms |  |  |  | 0.13^†^ |
| PBC |  |  |  | 0.18^*^ |
| Model 2 | 0.41 | 0.03 | 0.11 |  |
| Parent Status |  |  |  | -0.22^**^ |
| Affective Attitudes |  |  |  | 0.38^**^ |
| Instrumental Attitudes |  |  |  | 0.22^†^ |
| Subjective Norms |  |  |  | - 0.12 |
| PBC |  |  |  | 0.27^*^ |
| Affective Attitudes x Parent Status |  |  |  | <0.01 |
| Instrumental Attitudes x Parent Status |  |  |  | -0.17 |
| Subjective Norms x Parent Status |  |  |  | 0.32^*^ |
| PBC x Parent Status |  |  |  | -0.14 |
| Men (N = 145) |  |  |  |  |
| Model 1 | 0.54 | 0.53 | < 0.01 |  |
| Parent Status |  |  |  | -0.05 |
| Affective Attitudes |  |  |  | 0.55^**^ |
| Instrumental Attitudes |  |  |  | 0.09 |
| Subjective Norms |  |  |  | 0.16^*^ |
| PBC |  |  |  | 0.15^*^ |

Note. Model for male participants was adjusted for education. **p<.01, *p<.05, †p<.10

Table S3. Hierarchical Regression of TPB Variables Predicting Fruit & Vegetable Consumption for Women

| Six-Month Fruit & Vegetable (N=116) | R^2^ | R^2^Δ | *P* | β |  | 12-month Fruit & Vegetable (N=109) | R^2^ | R^2^Δ | *p* | β |
| --- | --- | --- | --- | --- | --- | --- | --- | --- | --- | --- |
| Model 1 | 0.17 | 0.18 | <.001 |  |  | Model 1 | 0.19 | 0.20 | <.01 |  |
| Baseline Fruit & Vegetable |  |  |  | 0.42^**^ |  | 6-month Fruit & Vegetable |  |  |  | 0.45^**^ |
| Model 2 | 0.17 | 0.02 | 0.51 |  |  | Model 2 | 0.18 | 0.02 | 0.56 |  |
| Baseline Fruit & Vegetable |  |  |  | 0.39^**^ |  | 6-month Fruit & Vegetable |  |  |  | 0.42^**^ |
| Parent Status |  |  |  | -0.03 |  | Parent Status |  |  |  | -0.02 |
| PBC |  |  |  | 0.11 |  | PBC |  |  |  | 0.14 |
| Intentions to Eat Healthy |  |  |  | 0.03 |  | Intentions to Eat Healthy |  |  |  | -0.01 |
| Model 3 | 0.16 | 0.01 | 0.70 |  |  | Model 3 | 0.17 | 0.01 | 0.68 |  |
| Baseline Fruit & Vegetable |  |  |  | 0.38^**^ |  | 6-month Fruit & Vegetable |  |  |  | 0.39^**^ |
| Parent Status |  |  |  | -0.02 |  | Parent Status |  |  |  | -0.02 |
| PBC |  |  |  | 0.10 |  | PBC |  |  |  | 0.15 |
| Intentions to Eat Healthy |  |  |  | 0.02 |  | Intentions to Eat Healthy |  |  |  | 0.02 |
| Affective Attitudes |  |  |  | 0.07 |  | Affective Attitudes |  |  |  | 0.01 |
| Instrumental Attitudes |  |  |  | 0.08 |  | Instrumental Attitudes |  |  |  | 0.02 |
| Subjective Norms |  |  |  | -0.08 |  | Subjective Norms |  |  |  | -0.12 |
| Model 4 | 0.21 | 0.06 | <.001 |  |  |  |  |  |  |  |
| Baseline Fruit & Vegetable |  |  |  | 0.42^**^ |  |  |  |  |  |  |
| Parent Status |  |  |  | -0.05 |  |  |  |  |  |  |
| PBC |  |  |  | 0.07 |  |  |  |  |  |  |
| Intentions to Eat Healthy |  |  |  | 0.03 |  |  |  |  |  |  |
| Affective Attitudes |  |  |  | 0.35^*^ |  |  |  |  |  |  |
| Instrumental Attitudes |  |  |  | 0.14 |  |  |  |  |  |  |
| Subjective Norms |  |  |  | -0.08 |  |  |  |  |  |  |
| Affective Attitudes x Parent Status |  |  |  | -0.40^*^ |  |  |  |  |  |  |

Note. **p<.01, *p<.05, †p<.10

Table S4. Hierarchical Regression of TPB Variables Predicting Fruit & Vegetable Consumption for Men

| Six-Month Fruit & Vegetable (N=119) | R^2^ | R^2^Δ | *p* | β |  | 12-month Fruit & Vegetable (N=101) | R^2^ | R^2^Δ | *p* | β |
| --- | --- | --- | --- | --- | --- | --- | --- | --- | --- | --- |
| Model 1 | 0.04 | 0.05 | 0.02 |  |  | Model 1 | 0.10 | 0.11 | <0.01 |  |
| Baseline Fruit & Vegetable |  |  |  | 0.22^*^ |  | 6-month Fruit & Vegetable |  |  |  | 0.33^**^ |
| Model 2 | 0.09 | 0.08 | 0.03 |  |  | Model 2 | 0.14 | 0.07 | 0.08 |  |
| Baseline Fruit & Vegetable |  |  |  | 0.18^†^ |  | 6-month Fruit & Vegetable |  |  |  | 0.30^**^ |
| Parent Status |  |  |  | <0.01 |  | Parent Status |  |  |  | -0.25^*^ |
| PBC |  |  |  | 0.22^*^ |  | PBC |  |  |  | -0.03 |
| Intentions to Eat Healthy |  |  |  | 0.13 |  | Intentions to Eat Healthy |  |  |  | 0.08 |
| Model 3 | 0.10 | 0.01 | 0.39 |  |  | Model 3 | 0.19 | 0.08 | 0.05 |  |
| Baseline Fruit & Vegetable |  |  |  | 0.19^*^ |  | 6-month Fruit & Vegetable |  |  |  | 0.38^*^ |
| Parent Status |  |  |  | 0.01 |  | Parent Status |  |  |  | -0.26^*^ |
| PBC |  |  |  | 0.21^*^ |  | PBC |  |  |  | -0.02 |
| Intentions to Eat Healthy |  |  |  | 0.17 |  | Intentions to Eat Healthy |  |  |  | 0.09 |
| Affective Attitudes |  |  |  | 0.09 |  | Affective Attitudes |  |  |  | -0.21^†^ |
| Instrumental Attitudes |  |  |  | -0.17 |  | Instrumental Attitudes |  |  |  | 0.25^*^ |
| Subjective Norms |  |  |  | -0.05 |  | Subjective Norms |  |  |  | -0.05 |
| Model 4 | 0.09 | 0.19 | 0.06 |  |  |  |  |  |  |  |
| Baseline Fruit & Vegetable |  |  |  | 0.20^*^ |  |  |  |  |  |  |
| Parent Status |  |  |  | 0.05 |  |  |  |  |  |  |
| PBC |  |  |  | 0.45^*^ |  |  |  |  |  |  |
| Intentions to Eat Healthy |  |  |  | 0.15 |  |  |  |  |  |  |
| Affective Attitudes |  |  |  | -0.01 |  |  |  |  |  |  |
| Instrumental Attitudes |  |  |  | 0.25 |  |  |  |  |  |  |
| Subjective Norms |  |  |  | -0.19 |  |  |  |  |  |  |
| PBC x Parent Status |  |  |  | -0.27 |  |  |  |  |  |  |
| Intentions x Parent Status |  |  |  | 0.05 |  |  |  |  |  |  |
| Affective Attitudes x Parent Status |  |  |  | 0.07 |  |  |  |  |  |  |
| Instrumental Attitudes x Parent Status |  |  |  | 0.06 |  |  |  |  |  |  |
| Subjective Norms x Parent Status |  |  |  | 0.20 |  |  |  |  |  |  |

Note. **p<.01, *p<.05, †p<.10

Table S5. Hierarchical Regression of TPB Variables Predicting Fat Consumption for Women

| Six-Month Fat Consumption (N=121) | R^2^ | R^2^Δ | *p* | β |  | 12-Month Fat Consumption (N=108) | R^2^ | R^2^Δ | *p* | β |
| --- | --- | --- | --- | --- | --- | --- | --- | --- | --- | --- |
| Model 1 | 0.05 | 0.05 | 0.01 |  |  | Model 1 | 0.12 | 0.13 | <0.001 |  |
| Baseline Fat |  |  |  | 0.23^**^ |  | 6-month Fat |  |  |  | 0.36^**^ |
| Model 2 | 0.05 | 0.03 | 0.34 |  |  | Model 2 | 0.14 | 0.05 | 0.17 |  |
| Baseline Fat |  |  |  | 0.20^*^ |  | 6-month Fat |  |  |  | 0.38^**^ |
| Parent Status |  |  |  | 0.15 |  | Parent Status |  |  |  | 0.10 |
| PBC |  |  |  | 0.06 |  | PBC |  |  |  | -0.23^*^ |
| Intentions to Eat Healthy |  |  |  | -0.05 |  | Intentions to Eat Healthy |  |  |  | 0.14 |
| Model 3 | 0.04 | 0.02 | 0.34 |  |  | Model 3 | 0.14 | 0.02 | 0.44 |  |
| Baseline Fat |  |  |  | 0.21^*^ |  | 6-month Fat |  |  |  | 0.38^**^ |
| Parent Status |  |  |  | 0.14 |  | Parent Status |  |  |  | 0.11 |
| PBC |  |  |  | 0.02 |  | PBC |  |  |  | -0.24^*^ |
| Intentions to Eat Healthy |  |  |  | -0.12 |  | Intentions to Eat Healthy |  |  |  | 0.17 |
| Affective Attitudes |  |  |  | 0.08 |  | Affective Attitudes |  |  |  | -0.13 |
| Instrumental Attitudes |  |  |  | 0.03 |  | Instrumental Attitudes |  |  |  | 0.11 |
| Subjective Norms |  |  |  | 0.10 |  | Subjective Norms |  |  |  | 0.07 |
| Model 4 | 0.07 | 0.03 | 0.05 |  |  |  |  |  |  |  |
| Baseline Fat |  |  |  | 0.19* |  |  |  |  |  |  |
| Parent Status |  |  |  | 0.12 |  |  |  |  |  |  |
| PBC |  |  |  | 0.22 |  |  |  |  |  |  |
| Intentions to Eat Healthy |  |  |  | -0.11 |  |  |  |  |  |  |
| Affective Attitudes |  |  |  | 0.07 |  |  |  |  |  |  |
| Instrumental Attitudes |  |  |  | 0.02 |  |  |  |  |  |  |
| Subjective Norms |  |  |  | 0.11 |  |  |  |  |  |  |
| PBC x Parent Status |  |  |  | -0.28^*^ |  |  |  |  |  |  |

Note. **p<.01, *p<.05, †p<.10

Table S6. Hierarchical Regression of TPB Variables Predicting Fat Consumption for Men

| Six-Month Fat Consumption (N=119) | R^2^ | R^2^Δ | *p* | β |  | 12-Month Fat Consumption (N=101) | R^2^ | R^2^Δ | *p* | Β |
| --- | --- | --- | --- | --- | --- | --- | --- | --- | --- | --- |
| Model 1 | 0.04 | 0.05 | 0.01 |  |  | Model 1 | 0.14 | 0.12 | <0.001 |  |
| Baseline Fat |  |  |  | 0.23^*^ |  | 6-month Fat |  |  |  | 0.35^**^ |
| Model 2 | 0.08 | 0.06 | 0.08 |  |  | Model 2 | 0.17 | 0.05 | 0.11 |  |
| Baseline Fat |  |  |  | 0.23^*^ |  | 6-month Fat |  |  |  | 0.38^**^ |
| Parent Status |  |  |  | 0.03 |  | Parent Status |  |  |  | 0.14 |
| PBC |  |  |  | 0.25^*^ |  | PBC |  |  |  | -0.24* |
| Intentions to Eat Healthy |  |  |  | -0.03 |  | Intentions to Eat Healthy |  |  |  | 0.11 |
| Model 3 | 0.06 | 0.01 | 0.74 |  |  | Model 3 | 0.16 | 0.01 | 0.66 |  |
| Baseline Fat |  |  |  | 0.24^**^ |  | 6-month Fat |  |  |  | 0.38^**^ |
| Parent Status |  |  |  | 0.04 |  | Parent Status |  |  |  | 0.15 |
| PBC |  |  |  | 0.27^**^ |  | PBC |  |  |  | -0.25^*^ |
| Intentions to Eat Healthy |  |  |  | 0.05 |  | Intentions to Eat Healthy |  |  |  | 0.12 |
| Affective Attitudes |  |  |  | -0.13 |  | Affective Attitudes |  |  |  | -0.08 |
| Instrumental Attitudes |  |  |  | 0.02 |  | Instrumental Attitudes |  |  |  | 0.07 |
| Subjective Norms |  |  |  | -0.05 |  | Subjective Norms |  |  |  | 0.09 |

Note. 12-Month model adjusted for participants’ education.
